# Supplementary material for: Antimicrobial Activity and Mode of Action of Celastrol, a Nortriterpen Quinone Isolated from Natural Sources
Source: Foods. 2021 Mar 11;10(3):591. doi: 10.3390/foods10030591 (PMC7998816; doi:10.3390/foods10030591)
Supplement: Supplementary file 1 [file foods-10-00591-s001.pdf]

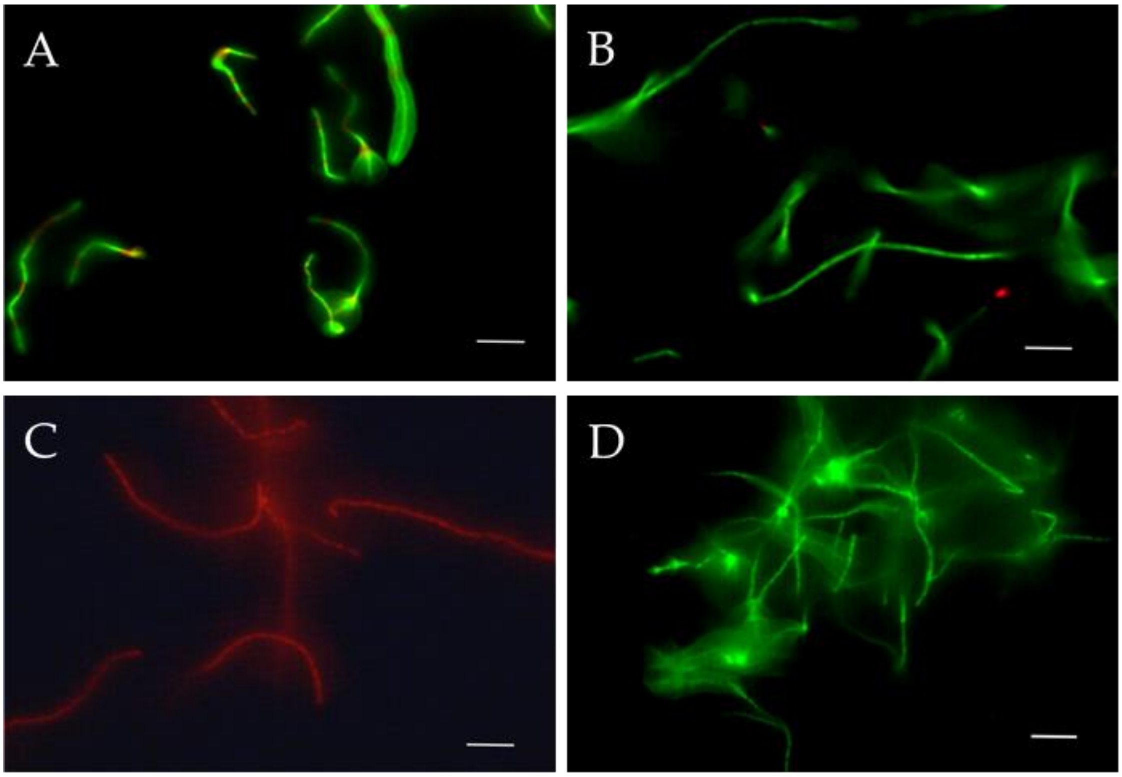

Figure S1. Epifluorescence microscopy images of *B. subtilis* stained with propidium iodide and SYTO 9 after treatment with celastrol (3 µg/mL) for 30 and 60 min (A, B) or clofoctol (5 µg/mL) for 30 min (C). Control cells were treated with the maximum proportion of DMSO (D). Scale bars correspond to 10 µm.

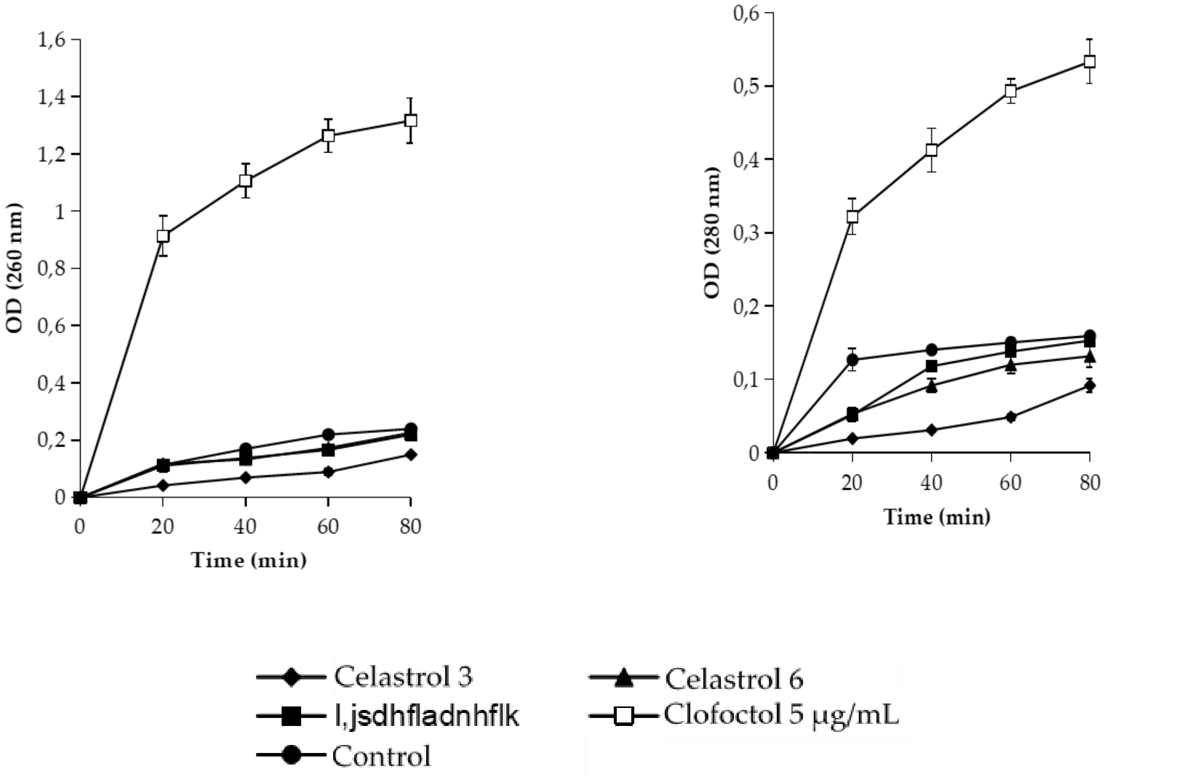

Figure S2. Release of 260 and 280 nm absorbing material from *B. subtilis* cells treated with celastrol at 3, 6, and 10 µg/mL and clofoctol at 5 µg/mL. Cells exposed to the maximum proportion of DMSO were used as control. Error bars express SD with n=3.

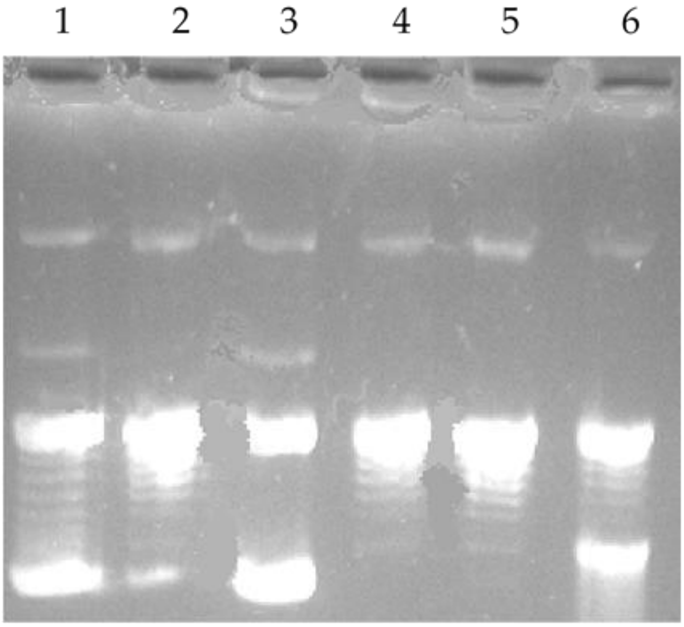

Figure S3. DNA supercoiling assays. Electrophoresis gel shows a control with a negatively supercoiled plasmid (Lane 1) with DMSO (line 2), and after its treatment with ciprofloxacin at 25 µg/mL (line 3) and 50 µg/mL (line 4), celastrol at 50 µg/mL (line 5), and pristimerin at 50 µg/mL (line 6).
